# Incidence and Estimated Prevalence of Endometriosis and Adenomyosis in Northeast Italy: A Data Linkage Study

**DOI:** 10.1371/journal.pone.0154227

**Published:** 2016-04-21

**Authors:** Caterina Morassutto, Lorenzo Monasta, Giuseppe Ricci, Fabio Barbone, Luca Ronfani

**Affiliations:** 1 Clinical Epidemiology and Public Health Research Unit, Institute for Maternal and Child Health–IRCCS “Burlo Garofolo”, Trieste, Italy; 2 Department of Obstetrics and Gynecology, Institute for Maternal and Child Health–IRCCS “Burlo Garofolo”, Trieste, Italy; 3 Department of Medicine, Surgery and Health Sciences, University of Trieste, Trieste, Italy; 4 Department of Medical and Biological Sciences, University of Udine, Udine, Italy; The Ohio State University, UNITED STATES

## Abstract

Despite being quite frequent and having serious implications in terms of symptomatology and fertility, data on incidence and prevalence of endometriosis and adenomyosis following gold standard definitions are dramatically lacking. The average time from onset of symptoms to diagnosis in industrialized countries still ranges from five to ten years. Using the regional centralized data linkage system, we calculated incidence and prevalence of endometriosis and adenomyosis in the female population of Friuli Venezia Giulia region, Italy, for the years 2011–2013. Cases were defined as new diagnoses from hospital discharge records, following procedures allowing direct visualization for endometriosis and hysterectomy for adenomyosis, with or without histological confirmation. Diagnoses were considered “new” after verifying women had not been diagnosed in the previous ten years. Incidence of endometriosis and adenomyosis in women aged 15–50 years is 0.14%. Prevalence, estimated from incidence, is 2.00%. Adenomyosis, representing 28% of all diagnoses, becomes increasingly prevalent after the age of 50 years. Our results shows how the study of both endometriosis and adenomyosis should not be limited to women of premenopausal age. Further efforts are needed to sensitize women and health professional, and to find new data linkage possibilities to identify undiagnosed cases.

## Introduction

Endometriosis and adenomyosis are gynecological diseases defined as the presence of endometrial tissue located in places other than physiologically appropriate. [1] Adenomyosis is defined as the presence of endometrial glands and stroma within the myometrium, whereas endometriosis is the presence of endometrial tissue outside the uterus. [2] Endometriosis and adenomyosis are steroid hormones dependent conditions and, as the normal endometrium, their development is regulated by the levels of estrogen and progesterone. [3]

Bleeding is one of the consequences of the response of the endometrial tissue to hormonal stimulation, and can lead to inflammation and scarring, and consequently to complications such as dysmenorrhea, infertility, chronic pelvic pain and dyspareunia. [4] Since the level of estrogen rapidly decreases around age 50, endometriosis and adenomyosis are considered to be a problem mainly before menopause. [3] However, there are reports on new cases identified beyond reproductive age since, for example, if iatrogenic or endogenous hormones are present, the disease can still be active. [1]

The public health burden of endometriosis and adenomyosis remains elusive, because the disease can be diagnosed accurately only by laparoscopy, laparotomy or hysterectomy, and magnetic resonance can be used only for lesions larger than 1 cm in diameter. In addition, many women are asymptomatic and some lesions might heal spontaneously without a diagnosis having been previously made. [5–7]

Despite the lack of reliable data, annual incidence estimates of endometriosis and adenomyosis jointly considered reported in population-based studies range from 0.1% to 0.2%, [4–6, 8, 9] while prevalence estimates of jointly considered endometriosis and adenomyosis based on representative samples of the general female population are rare, but range between 1.8% to 3.3%. [8, 10, 11] An ambiguity exists in literature on both incidence and prevalence estimates as often, even in studies clearly defining endometriosis as presence of endometrial tissue outside the uterine cavity, the authors present numbers including cases of adenomyosis. [4, 8, 9, 12]

In 2012 the first Italian law for the protection of women with endometriosis (including adenomyosis) was enacted in Friuli Venezia Giulia (FVG). As a consequence, a specific Regional Register of Endometriosis is being set up to monitor the disease, and define appropriate strategies. [13]

The aim of this study was to estimate the incidence and prevalence of endometriosis and adenomyosis during the years 2011–2013 in FVG using the Regional Register of Endometriosis, which is being created from the regional automated centralized record system.

## Materials and Methods

FVG is one of the 20 administrative regions of Italy. Located in the north east, it covers an area of 7855 Km^2^ and has a population of approximately 1.22 million people, of which 630 thousand are women. FVG is covered by an automated centralized record system developed in the 1980s with the goal of automatically pooling health care data from the national health service using a unique anonymous regional identification code. All data used in the present study were extracted from this regional epidemiological data warehouse.

Access to the data was granted by the Regional Health Authority that funded the study and the data we accessed were completely anonymized. For these reasons, no approval was required from the institutional review board of our Institute.

Data sources used for identifying newly diagnosed cases for the years 2011–2013 were hospital discharge records and anatomic pathology reports.

For the hospital discharge records we considered only women of 15 years of age or older residing in FVG with at least one hospitalization with a diagnosis of endometriosis or adenomyosis (*International Classification of Diseases*, *Ninth Revision* ICD-9, codes 617.0–617.9) in the years 2011–2013. The diagnosis of endometriosis had to be supported by laparoscopy or a similar surgical procedure allowing direct visualization, while the diagnosis of adenomyosis had to follow hysterectomy. Diagnoses only supported by imaging procedures (i.e. ultrasound, magnetic resonance, computerized axial tomography) were excluded from the count.

From the anatomic pathology records we selected, only for women of 15 years of age or older residing in the region, reports of both endometriosis and adenomyosis in the years 2011–2013.

Patients with a diagnosis of endometriosis or adenomyosis in the previous 10 years were excluded from the count of newly diagnosed cases.

We calculated incidence for the population of 15 to 50 years of age, and for the population of 15 years of age and older. For endometriosis, we followed the ESHRE (European Society of Human Reproduction and Embryology) guidelines for the gold standard diagnosis of endometriosis, defined as the combination of laparoscopy visualization and histologic confirmation of the presence of endometrial glands and/or stroma. [14, 15] For adenomyosis, the gold standard requires a positive histology following hysterectomy. [16, 17] For both age groups, we calculated two different incidence rates: one taking into account only positivity to histology, and the other considering a diagnosis in the inpatient records supported by laparoscopy or other surgical procedures for endometriosis and hysterectomy for adenomyosis, with or without positivity to histology. For the denominator, the number of women of 15 years of age or older residing in FVG was taken from the Italian National Institute of Statistics. [18] All incident cases were also separated into endometriosis and adenomyosis.

Prevalence was estimated from incidence, considering that endometriosis and adenomyosis most commonly affect women during their reproductive age and tend to decline after menopause because of the reduction in the production of estrogens. [19] Let us consider that (prevalence) = (incidence rate) x (average duration of disease), and reasonably assume that endometriosis and adenomyosis are chronic diseases that will last from diagnosis to at least menopause. This means that incident cases will keep cumulating until women reach menopause. We then decided to represent the situation of decline in prevalence after menopause under the simplifying hypothesis that menopause starts following a Gaussian curve with mean 51 years and 95% confidence interval of ±5 years (sd = 2.551). [20] To further simplify, we established that all women will enter menopause between 45 and 57 years of age. We thus reconverted the probabilities of the Gaussian to follow this last assumption. Finally, to represent the decline, not having any real data on which to base our decline in prevalence, we arbitrarily assumed that starting from age 45, every year 20% of women entering menopause will stop having the disease.

## Results

For the period 2011–2013, 1415 new cases of both endometriosis and adenomyosis were identified in the age range 15 to 83 (1017 and 398 respectively; Table 1): all cases of endometriosis had a laparoscopy or another surgical procedure allowing direct visualization, and a diagnosis of endometriosis, while all cases of adenomyosis had a hysterectomy and a diagnosis of adenomyosis in the inpatient record. Further 8 cases in the age range between 15 and 39 years of age were diagnosed following only imaging procedures and were thus excluded from the count. We also excluded 43 cases of adenomyosis not diagnosed with hysterectomy but only with direct visualization procedures.

**Table 1:** Age-specific incidence of endometriosis and adenomyosis in women residing in FVG in the years 2011–2013.

Age	Women residing in region*	Endometriosis and adenomyosis	Endometriosis	Adenomyosis
**15–20**	86938	11 (0.013%)	11 (0.013%)	0 (0.000%)
**21–25**	79379	40 (0.050%)	38 (0.048%)	2 (0.003%)
**26–30**	90201	116 (0.129%)	115 (0.127%)	1 (0.001%)
**31–35**	111355	201 (0.181%)	192 (0.172%)	9 (0.008%)
**36–40**	141918	218 (0.154%)	193 (0.136%)	25 (0.018%)
**41–45**	150254	262 (0.174%)	194 (0.129%)	68 (0.045%)
**46–50**	147005	268 (0.182%)	157 (0.107%)	111 (0.076%)
**51–55**	128019	113 (0.088%)	52 (0.041%)	61 (0.048%)
**56–60**	121556	55 (0.045%)	26 (0.021%)	29 (0.024%)
**61–65**	129679	53 (0.041%)	19 (0.015%)	34 (0.026%)
**66–70**	117903	30 (0.025%)	5 (0.004%)	25 (0.021%)
**71+**	370366	48 (0.013%)	15 (0.004%)	33 (0.009%)
**Total 15+**	1674573	1415 (0.084%)	1017 (0.061%)	398 (0.024%)
**Total 15–50**	807050	1116 (0.138%)	900 (0.112%)	216 (0.027%)

* Numbers represent the sum of women residing in the region in the three years considered.

Of the 1415 cases, 979 (69%) were histologically verified, that is 62% of endometrioses (626/1017) and 89% of adenomyoses (353/398) (Table 2). Considering only the age range 15–50, there were 1116 new cases and 719 of them had a positive histology (64%: 59% of endometrioses and 85% of adenomyoses).

**Table 2:** Age-specific incidence of histologically confirmed endometriosis and adenomyosis in women residing in FVG in the years 2011–2013.

Age	Women residing in region*	Endometriosis and adenomyosis	Endometriosis	Adenomyosis
**15–20**	86938	6 (0.007%)	6 (0.007%)	0 (0.000%)
**21–25**	79379	23 (0.029%)	23 (0.029%)	0 (0.000%)
**26–30**	90201	63 (0.070%)	62 (0.069%)	1 (0.001%)
**31–35**	111355	100 (0.090%)	93 (0.084%)	7 (0.006%)
**36–40**	141918	128 (0.090%)	108 (0.076%)	20 (0.014%)
**41–45**	150254	181 (0.120%)	124 (0.083%)	57 (0.038%)
**46–50**	147005	218 (0.148%)	119 (0.081%)	99 (0.067%)
**51–55**	128019	96 (0.075%)	38 (0.030%)	58 (0.045%)
**56–60**	121556	40 (0.033%)	17 (0.014%)	23 (0.019%)
**61–65**	129679	49 (0.038%)	17 (0.013%)	32 (0.025%)
**66–70**	117903	28 (0.024%)	5 (0.004%)	23 (0.020%)
**71+**	370366	47 (0.013%)	14 (0.004%)	33 (0.009%)
**Total 15+**	1674573	979 (0.058%)	626 (0.037%)	353 (0.021%)
**Total 15–50**	807050	719 (0.089%)	535 (0.066%)	184 (0.023%)

* Numbers represent the sum of women residing in the region in the three years considered.

**Fig 1:**
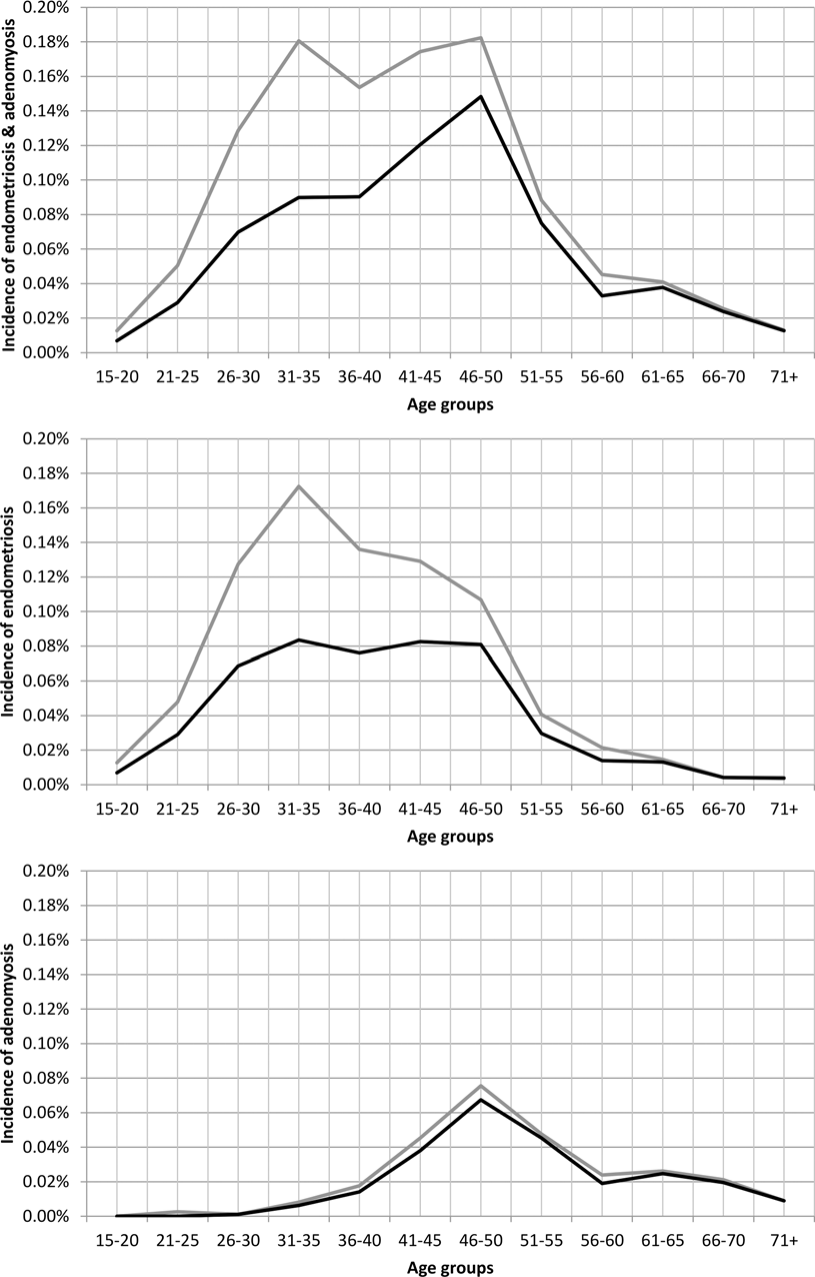
Age-specific incidence of endometriosis and adenomyosis in FVG in the years 2011–2013. The black line represents incidence of histologically confirmed diagnoses, while the grey line represents incidence of inpatient diagnoses supported by laparoscopy or another surgical procedure allowing direct visualization, regardless of the histological confirmation.

The crude incidence of endometriosis and adenomyosis in premenopausal women (15–50 years of age) for the period 2011–2013 was 0.14% if we consider all diagnoses with or without histological confirmation (Table 1). For histologically verified cases, the incidence in the same period was 0.09% (Table 2). The age-specific incidence of endometriosis was highest in the age group 31–35, while adenomyosis was highest in the age group 46–50 (Fig 1) (Tables 1 and 2). The incidence in women 15+ of jointly considered endometriosis and adenomyosis was 0.08% if we consider all diagnoses and 0.06 for histologically verified cases only (Tables 1 and 2).

Out of the 1415 new cases identified, 28% had a diagnosis of adenomyosis (Table 3). Adenomyosis becomes more prevalent that endometriosis after the age of 50 years.

**Table 3:** Cases of endometriosis and adenomyosis diagnosed in FVG in the years 2011–2013.

Age	Endometriosis	Adenomyosis	Total number of cases
**15–20**	11 (100%)	0 (0%)	11
**21–25**	38 (95%)	2 (5%)	40
**26–30**	115 (99%)	1 (1%)	116
**31–35**	192 (96%)	9 (4%)	201
**36–40**	193 (89%)	25 (11%)	218
**41–45**	194 (74%)	68 (26%)	262
**46–50**	157 (59%)	111 (41%)	268
**51–55**	52 (46%)	61 (54%)	113
**56–60**	26 (47%)	29 (53%)	55
**61–65**	19 (36%)	34 (64%)	53
**66–70**	5 (17%)	25 (83%)	30
**71+**	15 (31%)	33 (69%)	48
**Total 15+**	1017 (72%)	398 (28%)	1415
**Total 15–50**	900 (81%)	216 (19%)	1116

**Fig 2:**
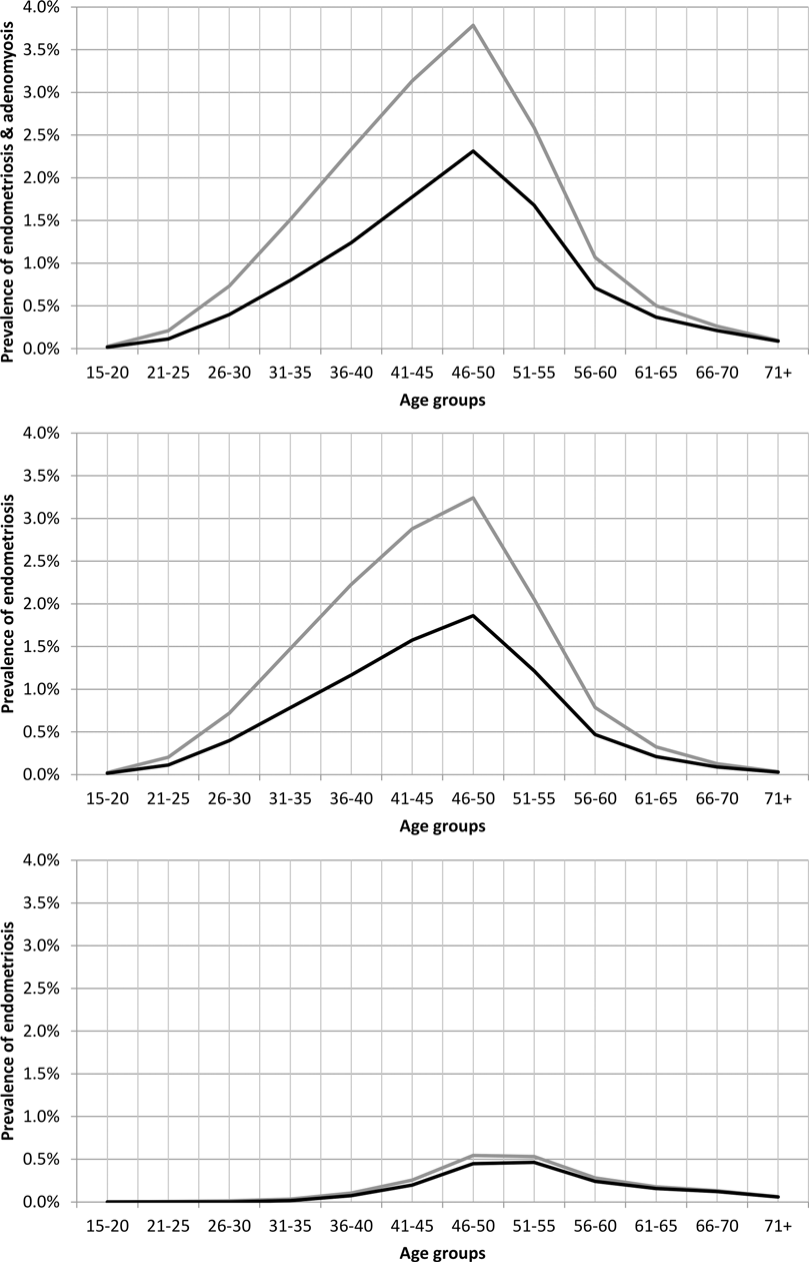
Age-specific prevalence of endometriosis and adenomyosis in FVG in the years 2011–2013. The black line represents the prevalence of diagnoses based on hysterectomy and histology, while the grey line represents the prevalence of diagnoses supported by hysterectomy, regardless of histological confirmation.

Prevalence of jointly considered endometriosis and adenomyosis was estimated from incidence, as specified in the Methods section, and for the age range 15–50 it was equal to 2.00% (Table 4, Fig 2): 1.82% for endometriosis and 0.17% for adenomyosis. Prevalence of only histologically confirmed diagnoses was 1.14% (1.00% and 0.13% respectively; Table 5, Fig 2). For the age range 15+, the prevalence of both endometriosis and adenomyosis was 1.32%, and 0.79% for histologically confirmed diagnoses only.

**Table 4:** Age-specific prevalence of endometriosis and adenomyosis in women residing in FVG in the years 2011–2013.

Age	Endometriosis and Adenomyosis	Endometriosis	Adenomyosis
**15–20**	0.02%	0.02%	0.00%
**21–25**	0.21%	0.20%	0.01%
**26–30**	0.73%	0.72%	0.01%
**31–35**	1.51%	1.48%	0.03%
**36–40**	2.33%	2.23%	0.10%
**41–45**	3.13%	2.88%	0.26%
**46–50**	3.79%	3.24%	0.54%
**51–55**	2.59%	2.06%	0.53%
**56–60**	1.07%	0.78%	0.28%
**61–65**	0.50%	0.32%	0.18%
**66–70**	0.26%	0.13%	0.13%
**71+**	0.09%	0.03%	0.06%
**TOTAL 15+**	1.32%	1.14%	0.18%
**TOTAL 15–50**	2.00%	1.82%	0.17%

**Table 5:** Age-specific prevalence histologically confirmed endometriosis and adenomyosis in women residing in FVG in the years 2011–2013.

Age	Endometriosis and Adenomyosis	Endometriosis	Adenomyosis
**15–20**	0.02%	0.02%	0.00%
**21–25**	0.11%	0.11%	0.00%
**26–30**	0.40%	0.40%	0.00%
**31–35**	0.80%	0.78%	0.01%
**36–40**	1.24%	1.16%	0.07%
**41–45**	1.77%	1.58%	0.20%
**46–50**	2.31%	1.86%	0.45%
**51–55**	1.68%	1.22%	0.46%
**56–60**	0.71%	0.47%	0.24%
**61–65**	0.37%	0.21%	0.16%
**66–70**	0.21%	0.09%	0.12%
**71+**	0.09%	0.03%	0.06%
**TOTAL 15+**	0.79%	0.64%	0.15%
**TOTAL 15–50**	1.14%	1.00%	0.13%

## Discussion

Our study shows an estimation of incidence and prevalence of endometriosis and adenomyosis consistent with those reported in population-based studies, [4–6, 8–11] even if comparisons between studies have limitations due to different settings and varying methodologies applied for case identification and definition. [5, 12] The strength of the present study is indeed in the possibility of linking detailed health information anonymously, relative to inpatient records (diagnoses, interventions and demographics), with the opportunity of following subjects back in time. Details on the type of interventions and on the localization of the endometrial tissue allowed us to unambiguously distinguish between endometriosis and adenomyosis and identify diagnoses based on direct visualization and positive histology.

Regarding incidence, if we limit the count to histologically confirmed new cases of jointly considered endometriosis and adenomyosis and to the population of women aged 15 to 50, according to our study we obtain 0.09%. For the same age group, if we also add the inpatient diagnoses with no histological confirmation, but following the appropriate surgical procedure allowing direct visualization, the incidence reaches 0.14%. As established by the ESHRE (European Society of Human Reproduction and Embryology) guidelines, the gold standard for the diagnosis of endometriosis is the combination of laparoscopy visualization and histologic confirmation of the presence of endometrial glands and/or stroma. [14, 15] The gold standard for the diagnosis of adenomyosis requires hysterectomy followed by histologic confirmation. [16, 17] Laparoscopy and hysterectomy, however, are invasive procedures and often other non-invasive techniques, such as ultrasound and magnetic resonance imaging (MRI), are preferred by clinicians for the identification of endometriosis and adenomyosis. [21] In fact, we found eight women in our records with an inpatient diagnosis confirmed by an imaging technique, and no surprise if all of these women were in their premenopausal age. An additional problem concerns the 43 women with a diagnosis of adenomyosis based on laparoscopy or similar procedures, which should not allow the correct visualization of the disease in the endometrium. [16, 17] Unfortunately, with our regional data linkage system we are unable at present to identify diagnoses done with medical imaging during an outpatient visit, and this aspect should be implemented in order to further investigate these cases. However, the sensitivity and specificity of ultrasound and MRI in the identification of endometriosis does not appear to be satisfactory. [14, 22] We believe this aspect should be further examined, as well as the existence of a tendency of proceeding towards a histological confirmation only in more serious cases. We are also aware of the fact that not all cases of endometriosis and adenomyosis end up been identified, for several reasons among which the lack or irrelevance of the symptoms, or the lack of association between symptoms and the disease, even by medical doctors. [5] The average time from onset of symptoms to diagnosis of endometriosis in industrialized countries is in the 5 to 10 years range. [23–26] In infertile women the delay in the diagnosis appears to be shorter than in women presenting pelvic pain. [23, 27] For this reason, we believe it is important to sensitize women and professionals, and to find new data linkage possibilities to identify potential cases.

Age is also an issue. Standard incidence is usually calculated on the population of women from 15 to 50 years of age, under the consideration that endometriosis and adenomyosis are diseases almost exclusively affecting women in their reproductive age. Symptoms are in fact strongly linked to the menstrual cycle, period in which the endometriotic tissue located outside the endometrium bleeds, causing swelling and inflammations. [1] However, from our data we notice that both endometriosis and adenomyosis do not simply disappear at age 50, and new diagnoses were registered up to 83 year of age. Indeed, despite the standard approach which considers that after menopause there is a tendency to atrophy and reabsorption of the endometriotic tissue, there are studies showing that endometrial deposits are still potentially active in older patients and can be reactivated in the presence of certain hormones. [2] Literature does not provide any definite evidence about the temporal course of neither endometriosis nor adenomyosis progression. Even though it is ascertained that endometriosis is a disease mainly occurring in women in their reproductive age, physicians should not exclude it in postmenopausal women in the presence of suggestive symptoms. [28] Our data show, after age 50, an increase in the proportion of cases of adenomyosis if compared to endometriosis. Adenomyosis, in fact, if compared to endometriosis, appears to be more frequent in adult women in their fourth and fifth decades. [29, 30] Moreover the mainstay of diagnosis and treatment of adenomyosis remains hysterectomy, and for its invasiveness such intervention tends to be more common after menopause, severity of symptoms being equal. [2] Anyway, it is worth noting that, depending on the age group, 17 to 47% of new diagnosis in postmenopausal women in our population were still related to endometriosis.

Over the last 30 years, several epidemiological studies have been conducted to determine the prevalence of endometriosis and adenomyosis. Most studies, however, have estimated the prevalence of endometriosis and adenomyosis in women with pelvic pain, infertility, and other gynecological conditions, [31] while only few have based they calculations on the general population. [8, 10, 11] A pooled analysis of published studies reported highly heterogeneous prevalence estimates of endometriosis and adenomyosis, this variation being attributable to the different methodologies applied for case identification and definition. [32] Actually, to underline how heterogeneity can affect the estimates, it is relevant to state how the authors of the pooled analysis fail to define endometriosis and to mention whether they include adenomyosis.

Our estimate for women 15–50 years of age ranges from 1.14% for only histologically confirmed cases to 2.00% for all visually supported diagnoses (laparoscopy or similar procedures for endometriosis and hysterectomy for adenomyosis), and can be compared to the one reported by Ferrero and colleagues (28/2000 = 1.4%) for women with a previous diagnosis of endometriosis or adenomyosis. [6] In the study by Ferrero the active search of the disease, using a questionnaire investigating the presence of typical symptoms of endometriosis/adenomyosis in an unselected sample of premenopausal women accessing their general practitioner because of non-gynecological problems, allowed to further identify and surgically confirm 37 cases, for an additional 1.85% prevalence. This result suggests that about 6 out of 10 cases were not identified before the active search of the disease. We believe that this might approximately be considered as the portion of relevant undiagnosed cases in the general population in this age range.
